# Saccadic reaction time and ocular findings in phenylketonuria

**DOI:** 10.1186/s13023-020-01407-7

**Published:** 2020-05-25

**Authors:** Susanne Hopf, Caroline Nowak, Julia B. Hennermann, Irene Schmidtmann, Norbert Pfeiffer, Susanne Pitz

**Affiliations:** 1 Department of Ophthalmology, University Medical Center Mainz, Langenbeckstr 1, 55131 Mainz, Germany; 2 Johannes Gutenberg University Mainz, Mainz, Germany; 3 Villa Metabolica, Department of Pediatric and Adolescent Medicine, University Medical Center Mainz, Mainz, Germany; 4 Institute of Medical Biostatistics, Epidemiology and Informatics (IMBEI), University Medical Center Mainz, Mainz, Germany; 5 Orbital Center, Ophthalmic Clinic, Bürgerhospital Frankfurt, Frankfurt, Germany

**Keywords:** Saccadometry, Video-oculography, Saccades, Phenylketonuria, Hyperphenylalaninemia, Phenylalanine hydroxylase deficiency, Aminoacid metabolism, Eye findings, Optical coherence tomography

## Abstract

**Background:**

Phenylketonuria (PKU) is an inherited metabolic disorder characterized by reduced activity of phenylalanine hydroxylase resulting in elevated blood phenylalanine (Phe) concentration. Despite some obvious ocular changes, the disorder has been poorly recognized by ophthalmologists. Neurophysiologic tests imply prolonged reaction time correlating with increased phenylalanine blood concentrations. We aimed to test saccadic reaction time in PKU patients in dependency of blood phenylalanine concentrations.

**Methods:**

Nineteen biochemically diagnosed PKU patients and 100 controls completed comprehensive ophthalmologic and orthoptic examinations including saccadometry by infrared based video-oculography. Peak velocity, gain, and particularly latency of reflexive saccades were compared to controls, and regression analysis was performed.

**Results:**

Latency of reflexive saccades was not associated with the current phenylalanine concentration. Although in 10 out of 19 patients phenylalanine concentrations were outside the age-related therapeutic range, latency differed little between PKU patients and the controls, as well as peak velocity and gain. Ocular findings occurred as partial hypopigmentation of the iris in one late diagnosed patient aged 36 years, and as bilateral cataracts (possibly due to steroid intake) with refractive amblyopia, strabismus, high myopia, and glaucoma in another late diagnosed patient aged 46 years. Visual acuity was reduced in eight PKU patients.

**Conclusions:**

Saccadometry, particularly saccadic reaction time, is not useful in the monitoring of phenylketonuria. Ophthalmic examination is recommended in PKU patients, as the occurrence of ocular pathologies was relatively high.

## Background

### Context and summary of the literature

Phenylketonuria (PKU) is an inherited disease of amino acid metabolism, with a prevalence of 1:5,352 in Germany (148 new cases in 2016) [1]. PKU is characterized by reduced activity of phenylalanine hydroxylase resulting in elevated blood phenylalanine (Phe) concentration. As the newborn screening on PKU is widely established, the timely initiation of a low phenylalanine diet prevents irreversible mental disability, and may enable affected patients to lead a near-normal life [2]. However, phenylalanine balanced diet becomes difficult in older children and adolescents, when they more often want to eat the same food as their friends and family [3]. The diet comprises a phenylalanine-free medical formula devoid of high protein food such as meat, fish, poultry, and measured amounts of fruits, vegetables, and low protein food (primarily vegan). Regular testing of phenylalanine blood concentrations is used to monitor the treatment.

Ocular findings in patients with phenylketonuria comprise a broad range of findings such as photophobia, ocular hypopigmentation, cataract, corneal clouding [4], subluxation of the lens [5], pallor of the optic disc and eventually even bilateral vision loss [6]. All of them occurred in rare cases.

Until now, there is no clear evidence that neurophysiologic tests on reaction time can be used as parameters for therapy monitoring [7]. Also, there is no tool for detecting phenylalanine affections of the brain [8]. Diamond and Herzberg hypothesized that executive function (prefrontal cortex of the brain) is depending on phenylalanine concentrations and that it is under dopaminergic control [9]. Therefore, we tested saccades as a potentially new parameter for therapy control, as saccades are under dopaminergic control, too [10, 11]. This would be in line with the ocular motor findings in other metabolic diseases than PKU such as Morbus Gaucher type 3 and Niemann Pick Type C, which show disturbances in horizontal and vertical saccadic eye movements, respectively [12, 13].

### Purpose of this study

The aim of this monocentric cohort study was the comprehensive analysis of saccade parameters, particularly saccadic latency (reaction time or delay) in patients with phenylketonuria under treatment. On the basis of reflexive saccades, we aimed to evaluate if saccadic latency could be a simple, non-invasive follow-up surrogate to the conventional phenylalanine blood test.

## Methods

This monocentric cohort study was conducted in 2015 in the University Medical Center Mainz in Germany. The study was approved by the Medical Ethical Committee of the State Chamber of Medicine of Rhineland Palatinate in Mainz, Germany (reference number 837.125.15). The patients or their parents/guardians gave a written consent to publication of their anonymized clinical data.

### Study participants

Inclusion criterion for patients was genetically and/or biochemically diagnosed phenylketonuria due to phenylalanine hydroxylase deficiency. Nineteen patients were included. A cohort of 100 healthy individuals at a stratified age distribution served as control [14]. For all participants, a visual acuity of better than (<) 1.3 logMAR at far distance was required, as well as age of at least 6 years, because at this age cooperation was regarded to be sufficient for the examination at that age.

### Examination procedure

The patients’ clinical data age at diagnosis, medication, current blood phenylalanine and tyrosine concentrations, and phenylalanine median of the year were obtained from their records.

All study participants received the following ophthalmologic examinations: refraction, best corrected visual acuity, anterior and posterior segment examination, Spectral Domain (SD)-OCT (Spectralis OCT, Heidelberg Engineering GmbH, Heidelberg, Germany. Module Heidelberg Eye Explorer 1.9.10.1, HRA2/ Spectralis Family Acquisition Module 6.0.11.0, HRA/ Spectralis Viewing Module 6.0.9.0) of the optic nerve head and the macula, orthoptic examinations (stereopsis, cover test, motility, optokinetic nystagmus and clinical saccade testing).

We performed a technical saccadometry using an infrared-videooculography (VOG) device named EyeSeeCam HIT (Eyeseetec, Fürstenfeldbruck), sampling at 220 Hz (every 3.6 ms) which was linked to a MacBook Pro 13″ (OS X Version 10.9.5, Apple Inc.) equipped with the EyeSeeCam software (EyeSeeCam VOG HIT System Reversion r3429 and r3444, EyeSeeTec Fürstenfeldbruck, Germany) [14].

Detection of saccades was at a threshold of 100°/s in peak velocity, beginning and ending at 5°/s, amplitude greater than 0.5 of the stimulus and latency not longer than 500 ms. Movements of the left eye were measured. The linear visual range was at 5°/15°/30° leftwards and rightwards and 5°/10°/20° upwards and downwards. Prior to the measurement, a qualitative calibration was performed. We performed a quality control of all raw data in the patient group (in the control group single artefacts would not have statistical impact). 125 out of 1924 (6.5%) saccades were excluded due to artefacts (see Additional file 1 A, and B), or anticipation being defined as covering about half of the distance prior to stimulus presentation (see Additional file 1 C), both shown in the additional file 1.

The saccade parameters we analyzed were latency (time interval between visual target and initiation of saccade, also called saccadic reaction time or delay, in the units milliseconds or the reciprocal term per second), peak velocity (maximal angular distance per time during the saccade), and gain (a dimension for precision, defined as quotient of amplitude and target displacement).

### Statistical analyses

Statistical analyses were performed with SPSS 23.0. Primary hypothesis was: Elevated blood phenylalanine concentrations have no impact on saccadic latency. Prior to analysis, a power calculation was performed to provide a power of over 80%. Patient characteristics are given as absolute and relative frequencies for categorical variables, and as mean and standards deviations for continuous variables.

We computed the reciprocal values of latency, with a target eccentricity of 15°, both to make the results comparable to previous studies. To test for the primary hypothesis, PKU patients with blood phenylalanine concentrations outside the age-related therapeutic range were compared to healthy controls with a student’s t-test. As the patients were younger than the control cohort (see Table 1), we compared them to only those controls aged under 50 years (*n* = 75).

**Table 1:** Characteristics of the study population

*Variable*	*Phenylketonuria patients n = 19*	*Controls n = 100*
Sex (female/male) [n/%]	15/4 (78.9%/21.1%)	54/46 (54.0%/46.0%)
Age [years]	20 SD 12 (Min. 6, Max. 46), 10 patients were < 18 years	33 SD 18 (Min. 6, Max. 75)
Blood Phe concentration [μmol/l]	782.72 SD 383.09 (Min. 210.60, Max. 1488.00), Median 694.20 -	–
Median yearly blood Phe concentration [μmol/l]	693.32 SD 383.79 (Min. 253.80, Max. 1425.00), Median 704.40	–
Blood tyrosine concentration [μmol/l]	104.48 SD 59.68 (Min. 37.53, Max. 215.79) Median 91.06	–

Bivariate Pearson’s correlation test was used with the variables latency and blood phenylalanine concentration. Correlation coefficients of *r* = 0.1, *r* = 0.3, and *r* = 0.5 were considered as weak, moderate, and strong, respectively [15]. We performed a linear multivariate regression analysis with current phenylalanine concentration as dependent variable and vertical and horizontal latencies as independent variables. For all analyses, a *p*-value of < 0.05 was considered statistically significant.

## Results

A detailed clinical table of each patient including ophthalmic laboratory data is given in the additional file 2. A summarized overview of the study population is listed in Table 1.

### Ophthalmologic findings in phenylketonuria

**Fig. 1:**
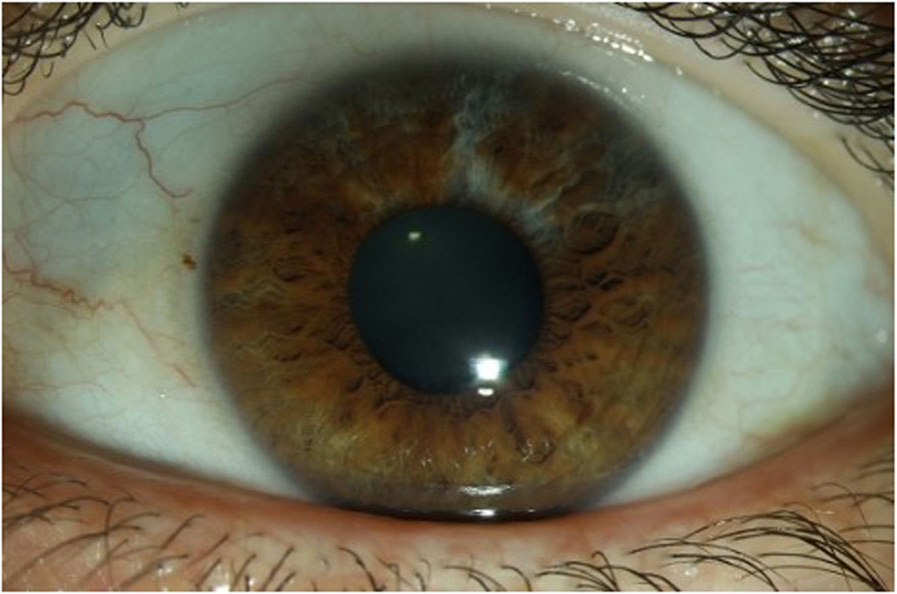
Arched hypopigmentation at 12 o’clock to the pupillary border of the iris to 2 o’clock in the left eye (the right eye showed normal pigmentation of the iris)

One patient with elevated blood phenylalanine concentration presented partial hypopigmentation of the iris (Fig. 1). This patient, aged 36 years, had not been diagnosed by newborn screening.

Two patients showed high myopia. One of them was missed by newborn screening and presented further eye diseases. These were amblyopia, strabismus, bilateral mild cataracts (possibly due to steroid intake), glaucoma, and retinal alterations, the latter being myopia-associated. In all patients, the OCTs of the macula and the optic nerve head were normal, apart from the optic discs of myopic patients.

Stereopsis was normal (40 arc sec) in 17/19 patients, and reduced in two patients (140 arc sec, and no stereopsis). Microstrabismus with mild amblyopia was the reason in one, and exotropia with moderate amblyopia in the other patient. Another six patients had slightly reduced visual acuity for unknown reason (three of them were children) (see Additional file 2).

Refraction (spherical equivalent) was classified as emmetropia in 15/38 (39%), mild myopia in 9/38 (24%), moderate or high myopia in 3/38 (8%) and hyperopia in 11/38 (29%) eyes. The distribution of refractive errors in our control group was roughly equivalent (49% emmetropic, 32% myopic, 1% high myopic and 19% hyperopic eyes).

### Results of the saccadometry

Reciprocal latency in patients with *abnormal* (above age-related therapeutic ranges) blood phenylalanine concentration was 5.29 1/s, while it was 5.65 1/s in the control group. The difference of the mean was 12 ms (189 ms in PKU vs. 177 ms in controls), which was neither significant (*p* = 0.318), nor clinically relevant.

Reciprocal latency in patients with *normal* (within age-related therapeutic ranges) blood phenylalanine concentrations was 5.57 1/s, and therefore not different to controls (*p* = 0.808). The mean latency was 180 ms, which was 3 ms longer than in controls (Table 2).

**Table 2:** Latency in phenylketonuria patients and in controls

*Study participants*	*Reciprocal Latency (horizontally)*	*Latency (horizontally)*
PKU all (*n* = 19)		
Phenylalanine concentrations above age-related therapeutic ranges (*) (*n* = 10)	5.291/s (SD 0.99)	189 ms
Phenylalanine concentrations within age-related therapeutic ranges (*n* = 9)	5.75 1/s (SD 1.05)	180 ms
Controls (*n* = 75)	5.65 1/s (SD 1.05)	177 ms

*: 238.4 μmol/l (64%) higher in comparison to the maximal age adjusted reference

The results for the reported horizontal saccades were similar to the results of vertical saccades.

**Fig. 2:**
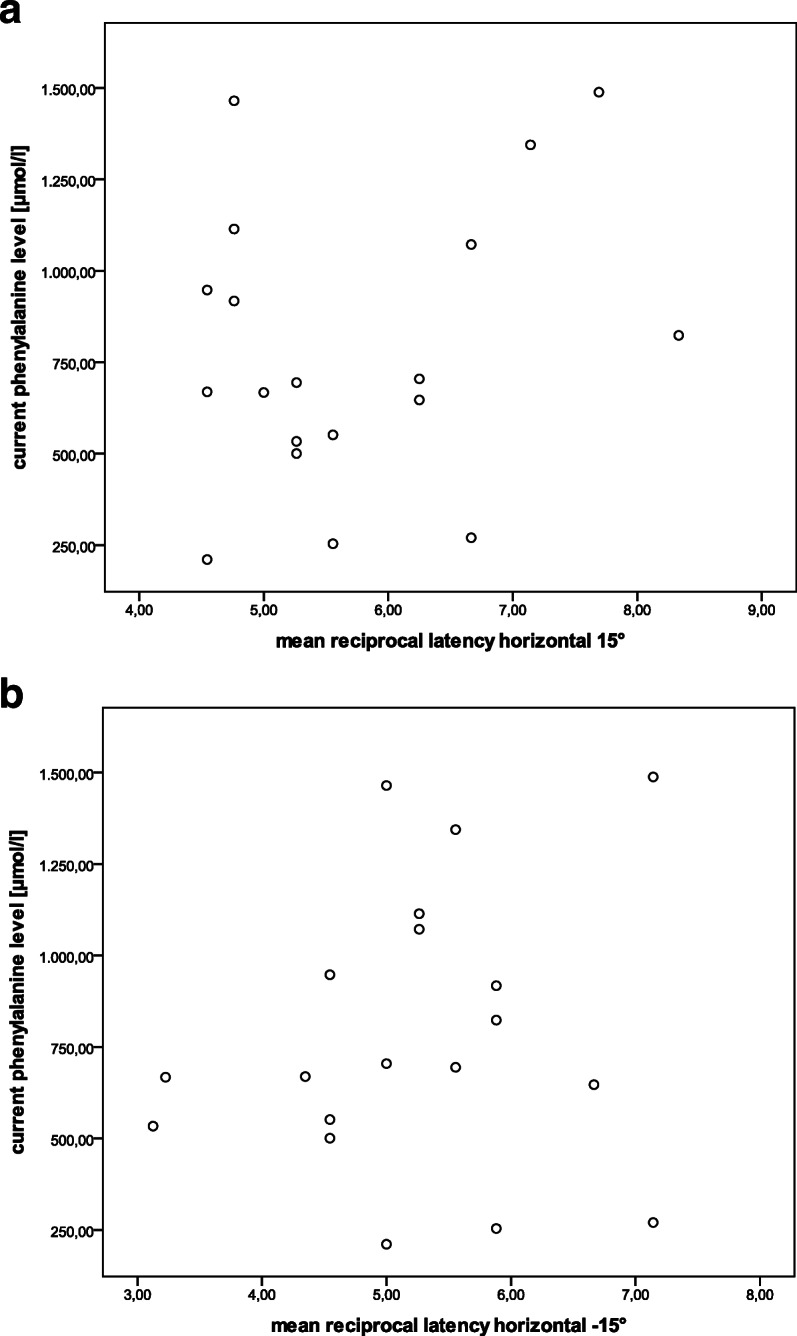
Scatter plots with reciprocal latency on the y-axis and current phenylalanine concentration on the x-axis (**a** saccades 15° rightwards, **b** 15° leftwards)

Pearson correlation analysis revealed weak correlation of reciprocal latency horizontally with the current phenylalanine concentration (*r* = 0.159; *p* = 0.515 and *r* = 0.252; *p* = 0.298 for horizontal 15° saccades, see Fig. 2a and b), whereas this was not reproducible for vertical saccades and for horizontal and vertical saccades together.

Linear multivariate regression with current blood phenylalanine concentration as dependent variable, and vertical and horizontal latencies as independent variables, showed that an increase of the phenylalanine concentration of 16.83 μmol/l was associated with a shorter latency of 1 ms (*p* = 0.036) horizontally, whereas for vertical saccades, increase of phenylalanine concentration of 11.53 μmol/l was associated with a longer latency (*p* = 0.065) (see Additional file 3). This was not estimated to have clinical relevance.

Peak velocity was faster in patients with phenylketonuria with a mean of 352.11 °/s (SD 33.48) compared to controls who presented a peak velocity of 339.80 °/s (SD 42.32), *p* = 0.234. The results for horizontal saccades as reported, were similar to those for vertical saccades (mean of 311.15 °/s in patients vs. 300.38 °/s in controls; *p* = 0.312). The results are shown in Table 3.

**Table 3:** Peak velocity (mean and standard deviation) in phenylketonuria patients and in controls

*Study participants*	*Horizontal saccades*	*Vertical saccades*
PKU all (*n* = 19)	352.11/s (SD 33.48)	311.15 °/s (SD 36.67)
Phenylalanine concentrations within age-related therapeutic ranges (*n* = 9)	353.61°/s (SD 24.42)	316.02°/s (SD 42.60)
Phenylalanine concentrations above age-related therapeutic ranges (*) (*n* = 10)	350.77°/s (SD 39.57)	306.77 °/s (SD 33.25)
Controls (*n* = 99)	339.80 °/s (SD 42.32)	300.38 °/s (SD 43.28)

(*): 238.4 μmol/l (64%) higher in comparison to the maximal age adjusted reference

Peak velocity increased with increasing stimulus in both groups (see Additional file 4).

Saccades were mostly isometric or slightly hypometric; and the vertical saccades were more hypometric than the horizontal saccades (see Additional file 5). Both findings were similar in the controls.

## Discussion

To the best of our knowledge, our study is the first comprehensive ophthalmologic and saccadometric analysis of children and adults suffering from PKU. We aimed to find out whether saccadometric parameters - particularly latency of reflexive saccades - are valuable as a surrogate for monitoring dietary treatment of phenylketonuria as proposed by a previous study [16]. The second aim was to comprehensively describe ophthalmologic findings in PKU including up to date imaging such as optical coherence tomography.

In our PKU cohort, saccadic latency was not correlated with blood phenylalanine concentrations. Therefore we do not regard latency useful as a surrogate for phenylalanine concentrations or treatment response. In contrast, Dawson et al. reported elevated phenylalanine concentrations to correlate with longer latency in adult PKU patients. Latency was 12 ms longer in PKU patients than in controls in both Dawson et al.’s, and our study. However, this result was not significant in our cohort. Our protocol provided 12 stimuli, of which six were presented horizontally and vertically each, making a bias due to few values less probable than in the study from Dawson et al., who only used three stimuli in horizontal direction at one target eccentricity.

Albrecht et al. conducted a meta-analysis on various neurophysiological reaction tests in patients with phenylketonuria. They reported an overall increase of reaction time compared to healthy controls. Most studies included in this meta-analysis measured choice reaction time, which measures the speed of decision-making [7]. Saccadic latency potentially reflects information about decision processes [17] (depending on the test method). Thus we cannot exclude an association. For reflexive short-latency saccades however, as tested in our study, we could not find an association to phenylalanine concentrations. Therefore, the superior colliculus which is normally under tight control from cortical areas, responsible for the generation of reflexive short-latency saccades [17] is probably not affected.

Most of the patients in our study revealed normal anatomical eye findings except for a single hypopigmentation of the iris in one patient, and another patient with cataracts (possibly steroid-induced cataracts) at the age of 46 with further abnormalities such as refractive amblyopia, strabismus, high myopia and glaucoma. On one hand, both patients were not diagnosed by newborn screening. On the other hand, there was a third study participant with missed newborn screening, who presented normal eye findings. Our results are in line with the few case reports on eye findings in patients with phenylketonuria during the twentieth century [4–6], in which cataract development was not directly related to PKU, but rather due to blunt trauma (often self-induced, and unilaterally), or due to high dosages of thioridazine (bilateral cataract) [4, 18]. Before the era of the newborn screening however, deficient pigmentation leading to blond hair and pale blue irides was common, with photophobia as a frequent result. In hypopigmented albinos, abnormal visual routing of nerve fibers can be found, while this is not true for PKU patients [4, 19]. We additionally found no fovea hypoplasia in the macula OCT in PKU.

### Strengths and limitations

One strength of this study was the consideration of children and teenagers, they are known to have difficulties in following their phenylalanine balanced diet [20]. There is a possible selection bias in the reseults due to the inhomogeneity of the sample regarding sex and regarding the severity of phenylketonuria, as well as by the small sample size. The latter being one reason why significant results might have been undetected.

## Conclusion

Although there was a minimal difference in saccadic latency between PKU patients with elevated phenylalanine concentrations and controls, longer latencies were not associated with phenylalanine concentrations. The oculomotor system is not impaired by elevated phenylalanine concentrations in the blood. Therefore, we cannot recommend to use this type of reaction time as surrogate for monitoring phenylalanine concentrations or treatment success in phenylketonuria.

Ocular findings in phenylketonuria may involve iris and lens abnormalities in rare cases, and also amblyopia due to refractive errors and strabismus especially. In our sample the prevalence of ocular findings was higher in patients who did not undergo newborn screening. However they are probably not directly related. We recommend ophthalmic examination in young children with phenylketonuria, especially if the newborn screening was not performed, or if diet regime was not adhered to.

## Supplementary information

**Additional file 1.** Artefacts in saccadic detection (Figures A, B, and C).

**Additional file 2.** Clinical data of all PKU patients with the main ophthalmologic and saccadometric findings, and blood values (Table).

**Additional file 3.** ANOVA and regression analysis with current phenylalanine level as dependent variable and horizontal and vertical latencies [ms] as independent variable (Table).

**Additional file 4.** Boxplots of peak velocity in PKU patients and controls (Figure).

**Additional file 5.** Boxplots of gain (accuracy) in PKU patients and controls (Figure).

## Data Availability

Data generated or analyzed during this study are included in this published article.

## Abbreviations

PKU: Phenylketonuria
Phe: Phenylalanine
SD-OCT: Spectral domain optical coherence tomography
