# Supplementary material for: Saccadic reaction time and ocular findings in phenylketonuria
Source: Orphanet J Rare Dis. 2020 May 25;15:124. doi: 10.1186/s13023-020-01407-7 (PMC7249436; doi:10.1186/s13023-020-01407-7)
Supplement: Supplementary file 1 — Additional file 1. Artefacts in saccadic detection (Figures A, B, and C). [file 13023_2020_1407_MOESM1_ESM.docx]

**Additional file 1: A, B, C, D: Artefacts in saccadic detection**

*A: Blinking artefacts*

**
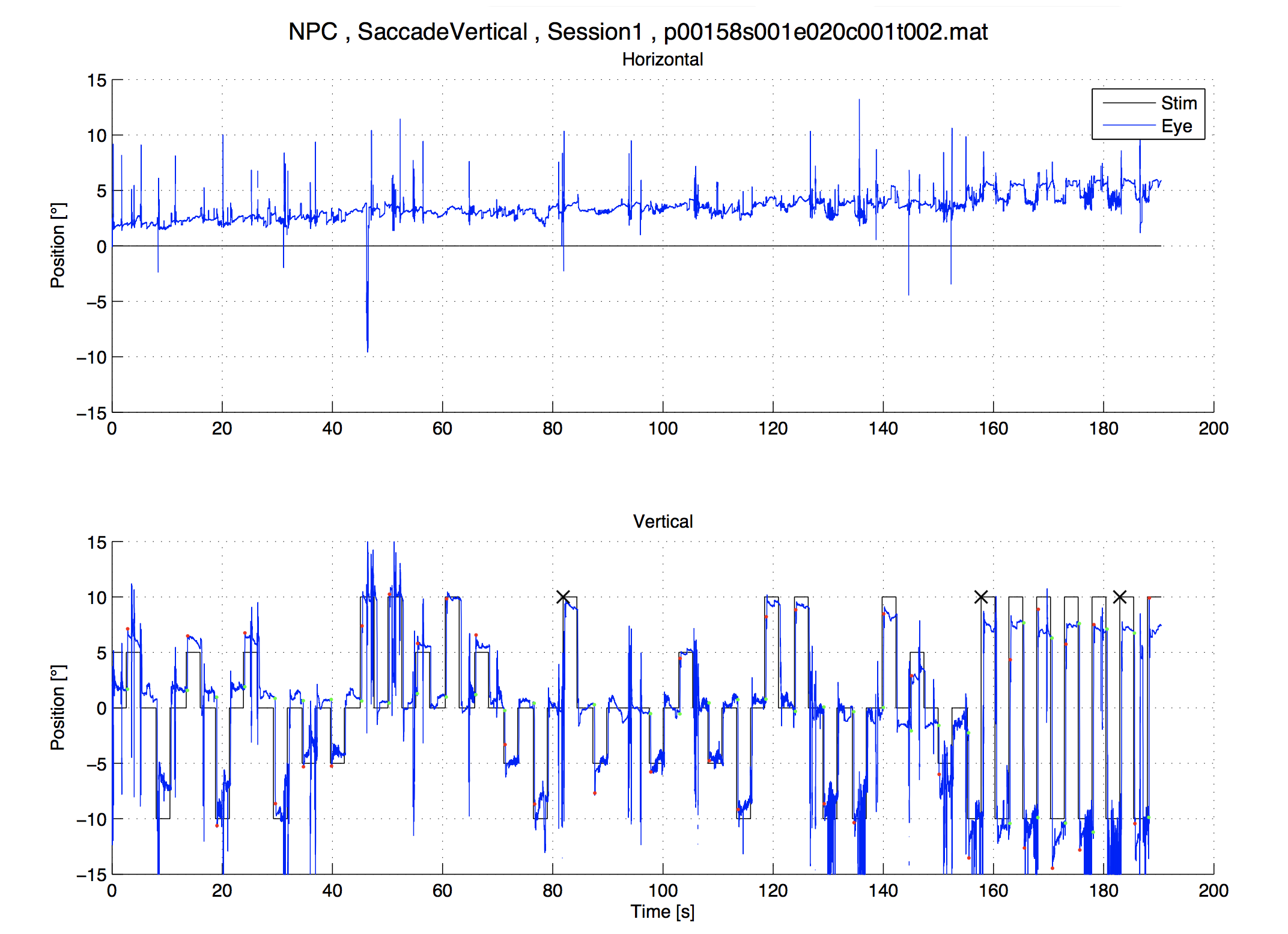
**

*Single values were excluded (e.g. between time 80 and 100, indicated by a cross in the stimulus trace), if a saccade was recorded (green starting point, and red ending point) while substantial blinking was present. This could be obvious by large runaways in the eye traces (blue lines).*

*B: Interruption of saccade (or in combination with missing start-/endpoints)* **
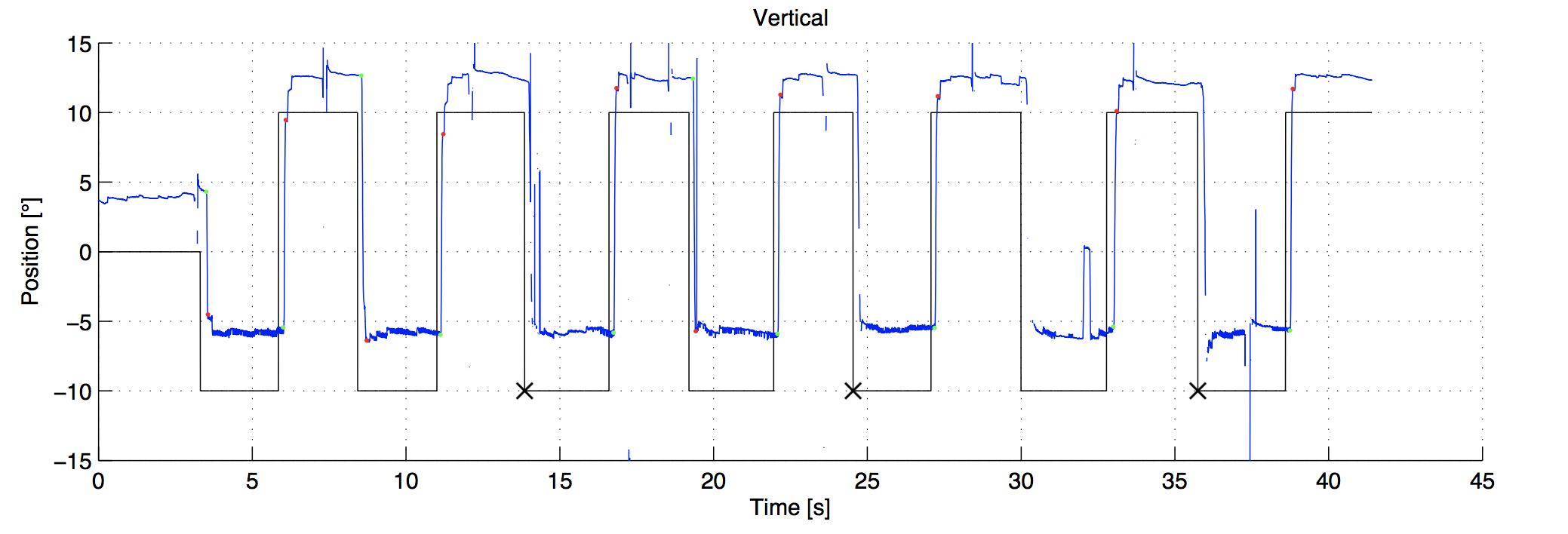
**

*If the eye trace (blue line) of a recorded saccade (defined by a green starting point, and a red ending point) was interrupted, single saccades were excluded (15th, 25th, and 35th sec.). If such an interrupted saccade was not recorded as a saccade, exclusion was not necessary (30Th sec.). In the figure, the difference is not seen, because the points disappear, when deleting is performed (cross in the stimulus trace).*

*C: Anticipation*

**
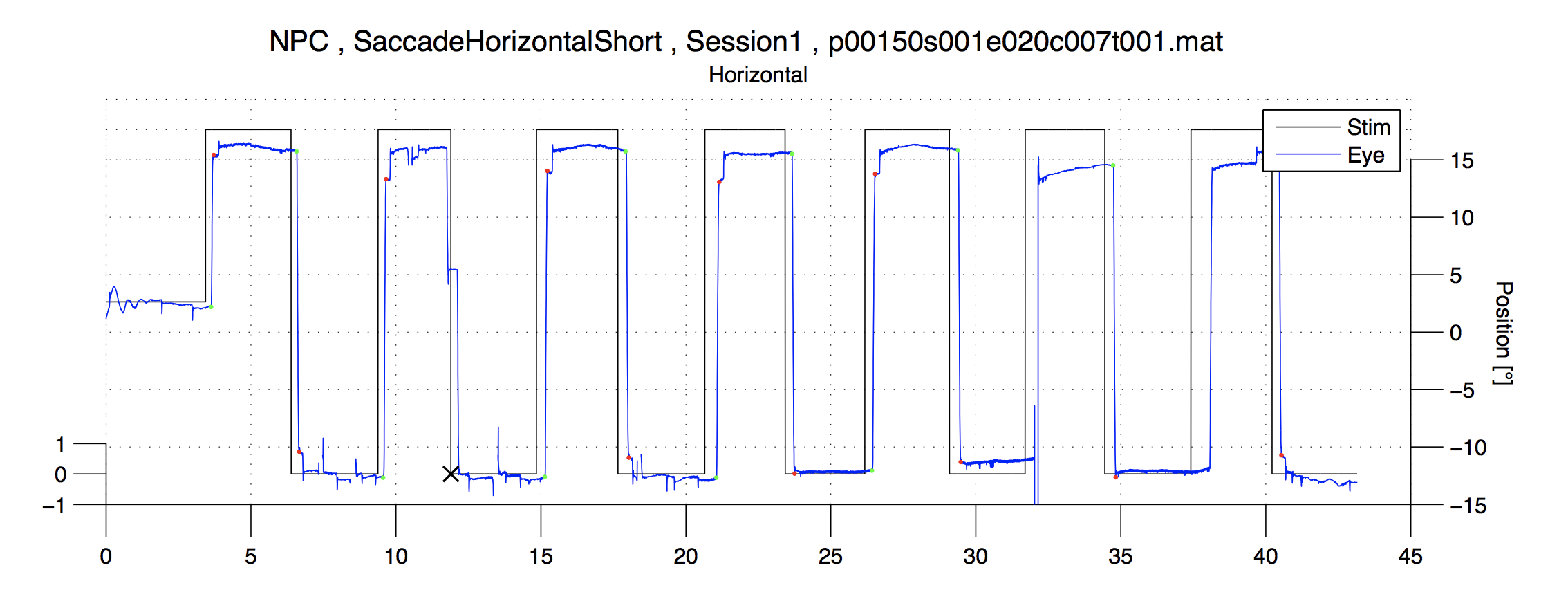
**

*Anticipation defined as covering about half of the distance prior to stimulus presentation (12th sec.).*
