# Supplementary material for: Saccadic reaction time and ocular findings in phenylketonuria
Source: Orphanet J Rare Dis. 2020 May 25;15:124. doi: 10.1186/s13023-020-01407-7 (PMC7249436; doi:10.1186/s13023-020-01407-7)
Supplement: Supplementary file 2 — Additional file 2. Clinical data of all PKU patients with the main ophthalmologic and saccadometric findings, and blood values (Table). [file 13023_2020_1407_MOESM2_ESM.docx]

**Additional file 2: Clinical data of all patients**

*Table: Clinical data of all PKU patients with the main ophthalmologic and saccadometric findings, and blood values.*

| *ID* | *Disease severity* | *Newborn screening* | *Age (years)* | *Sex* | *Age group* | *Medication* | *Ocular findings* | *Best distance visual acuity (decimal)* | *Refraction (sphere)* | *Blood phenylalanine concentration (µmol/l)* | *Blood tyrosine concentration (µmol/l)* | *Latency (sec.) rightwards*  *and*  *upwards* | | *Gain*  *rightwards*  *and*  *upwards* | | |  |
| --- | --- | --- | --- | --- | --- | --- | --- | --- | --- | --- | --- | --- | --- | --- | --- | --- | --- |
| 150 | classic | No | 36 | F | 2 | Y (*1) | Sectorial hypopigmentation of the iris OS | 0.8 / 0.63 | -1.5/ -1.25 | 1,464.60 | 184.89 | 0.21 | 0.20 | | 0.83 | 0.91 | |
| 153 | classic | Y | 8 | F | 0 | No | Normal | 1 / 0.8 | -1.25 / -1.0 | 669.00 | 96.58 | 0.22 | 0.16 | | 0.90 | 0.93 | |
| 154 | BH-4 | Y | 13 | F | 1 | Y (*2) | Normal | 1 / 1 | 0 / -0.25 | 270.00 | 61.81 | 0.15 | 0.14 | | 0.79 | 0.85 | |
| 156 | classic | Y | 26 | F | 2 | Y (*3) | Normal | 1 / 1 | +1.25 / +0.75 | 823.20 | 184.34 | 0.12 | 0.14 | | 0.75 | 0.83 | |
| 157 | classic | No | 22 | M | 2 | No | Normal | 1 / 1 | 0 / 0 | 1,488.00 | 37.53 | 0.13 | 0.14 | | 0.88 | 0.71 | |
| 158 | classic | Y | 8 | M | 0 | No | Titmus Rings No. 4, microstrabismm mild amblyopia | 0.8 / 0.8 | +1.0 /+ 1.0 | 533.40 | 52.43 | 0.19 | 0.21 | | 0.86 | 0.94 | |
| 159 | classic | Y | 8 | M | 0 | No | Normal | 0.8 / 0.8 | 0 / 0 | 500.40 | 61.81 | 0.19 | 0.22 | | 0.89 | 0.99 | |
| 160 | classic | Y | 10 | F | 0 | No | Normal | 1 / 1 | +0.25 / 0 | 1,114.20 | 44.70 | 0.21 | 0.19 | | 0.96 | 0.90 | |
| 161 | classic | Y | 6 | F | 0 | No | Normal | 0.8 / 1 | +0.75 / +0.75 | 210.60 | 215.79 | 0.22 | 0.19 | | 0.83 | 0.91 | |
| 162 | BH4 | Y | 27 | F | 2 | No | Normal | 1 / 1 | +1.0 / +0.75 | 253.80 | 91.06 | 0.18 | 0.16 | | 0.80 | 0.89 | |
| 163 | mild | Y | 18 | M | 2 | No | Normal | 1.6 / 1.25 | +0.5 / +0.5 | 646.80 | 80.58 | 0.16 | 0.17 | | 0.89 | 0.99 | |
| 164 | classic | Y | 26 | F | 1 | Y (*4) | Normal | 1.25 / 1.25 | 0 / 0 | 1,344.00 | 142.94 | 0.14 | 0.18 | | 0.83 | 0.91 | |
| 165 | classic | Y | 40 | F | 2 | No | High/moderate myopia OD/OS, optic discs tilted OD/OS | 1 / 1 | -7.25 / -4.0 | 947.40 | 48.57 | 0.22 | 0.20 | | 0.91 | 0.92 | |
| 166 | classic | Y | 13 | F | 2 | No | Mild Ptosis OS | 1 / 1 | -0.5 / -0.25 | 1,071.60 | 91.62 | 0.15 | 0.14 | | 0.73 | 0.99 | |
| 167 | classic | Y | 8 | F | 0 | No | Normal | 1 / 1 | 0 / +0.75 | 917.40 | 202.55 | 0.21 | 0.19 | | 0.82 | 0.85 | |
| 169 | classic | Y | 23 | F | 2 | Y (*5) | Peripheral retinal degeneration OD | 0.63 / 1 | -1.0 / -1.0 | 704.40 | 70.09 | 0.16 | 0.25 | | 0.87 | 1.02 | |
| 170 | classic | Y | 36 | F | 2 | Y (*6) | Normal | 0.8 / 1 | -1.0 / -0.75 | 694.20 | 91.62 | 0.19 | 0.19 | | 0.91 | 0.81 | |
| 171 | classic | Y | 9 | F | 0 | No | Normal | 1 / 1 | 0 / +0.25 | 551.40 | 175.51 | 0.18 | 0.19 | | 0.81 | 0.91 | |
| 172 | classic | No* | 46 | F | 2 | Y (*7) | High myopia OD, exotropia OD, no Stereopsis in Titmus fly and ring test, moderate amblyopia, glaucoma, mild cataracts + myopic optic discs + stretched maculae and retinae OD/OS | 0.32 / 0.63 | -6.5 / +0.25 | 667.20 | 50.78 | 0.20 | 0.27 | | 0.76 | 0.90 | |
| n=19 | 16x classic 1x mild 2x BH-4 | No/Yes 2/17 | 6 to 46 | M/F 4/15 | 7x0 2x1 10x2 | No/Yes 12/7 |  |  | 15x emmetropia 9x myopia 3x moderate or high myopia 11x hyperopia |  |  |  |  | |  |  | |

* patient lives in an assisted living and is equipped with a walker-rollator, (*1) neuroleptic (pipamperone, quentiapin), (*2) sapropterinhydrochloride (comparable to BH4 (setrahydrobiopterin), no phe restricted diet), (*3) co-trimoxazole, contraceptive, (*4 and *5) contraceptives, (*6) duloxetine, L-thyroxine, (*7) L-thyroxine, pantoprazole, beclometasone, montelukast, cholecalciferol antiglaucomatous eye drops: brimonidine, dorzolamide, bimatoprost.
