# Supplementary material for: Saccadic reaction time and ocular findings in phenylketonuria
Source: Orphanet J Rare Dis. 2020 May 25;15:124. doi: 10.1186/s13023-020-01407-7 (PMC7249436; doi:10.1186/s13023-020-01407-7)
Supplement: Supplementary file 3 — Additional file 3. ANOVA and regression analysis with current phenylalanine level as dependent variable and horizontal and vertical latencies [ms] as independent variable (Table). [file 13023_2020_1407_MOESM3_ESM.docx]

**Additional file 3: ANOVA and regression analysis**

Table: ANOVA and regression analysis with current phenylalanine concentration as dependent variable and horizontal and vertical latencies [ms] as independent variable.

| **ANOVA^a^** | | | | | | | | |
| --- | --- | --- | --- | --- | --- | --- | --- | --- |
| Model | | | Squared sum | | df | Mean squared error (MSE) | F | Sig. |
| 1 | | Regression | 654872.032 | | 2 | 327436.016 | 2.637 | .102^b^ |
|  |  | Non standardized residuals | 1986825.513 | | 16 | 124176.595 |  |  |
|  |  | Sum | 2641697.545 | | 18 |  |  |  |
| a. Dependent variable: current phenylalanine concentration [μmol/l]  b. Independent variables: (constant), mean of all vertical latencies [ms], mean of all horizontal latencies [ms] | | | | | | | | |
| **Coefficients^a^** | | | | | | | | |
|  | | | | Non standardized coefficients | | Standardized coefficients | T | Sig. |
|  |  |  |  | Regression Coefficent | Standard error | Beta |  |  |
| 1 | (Constant) | | | 1638.934 | 562.421 |  | 2.914 | .010 |
|  | Mean of all horizontal latencies [ms] | | | -16.829 | 7.361 | -1.170 | -2.286 | .036 |
|  | Mean of all vertical latencies [ms] | | | 11.532 | 5.827 | 1.013 | 1.979 | .065 |
| 1. Dependent variable: current phenylalanine concentration [μmol/l] | | | | | | | | |
