# Supplementary material for: Saccadic reaction time and ocular findings in phenylketonuria
Source: Orphanet J Rare Dis. 2020 May 25;15:124. doi: 10.1186/s13023-020-01407-7 (PMC7249436; doi:10.1186/s13023-020-01407-7)
Supplement: Supplementary file 4 — Additional file 4. Boxplots of peak velocity in PKU patients and controls (Figure). [file 13023_2020_1407_MOESM4_ESM.docx]

**Additional file 4: Boxplots of peak velocity in PKU patients and controls**

**
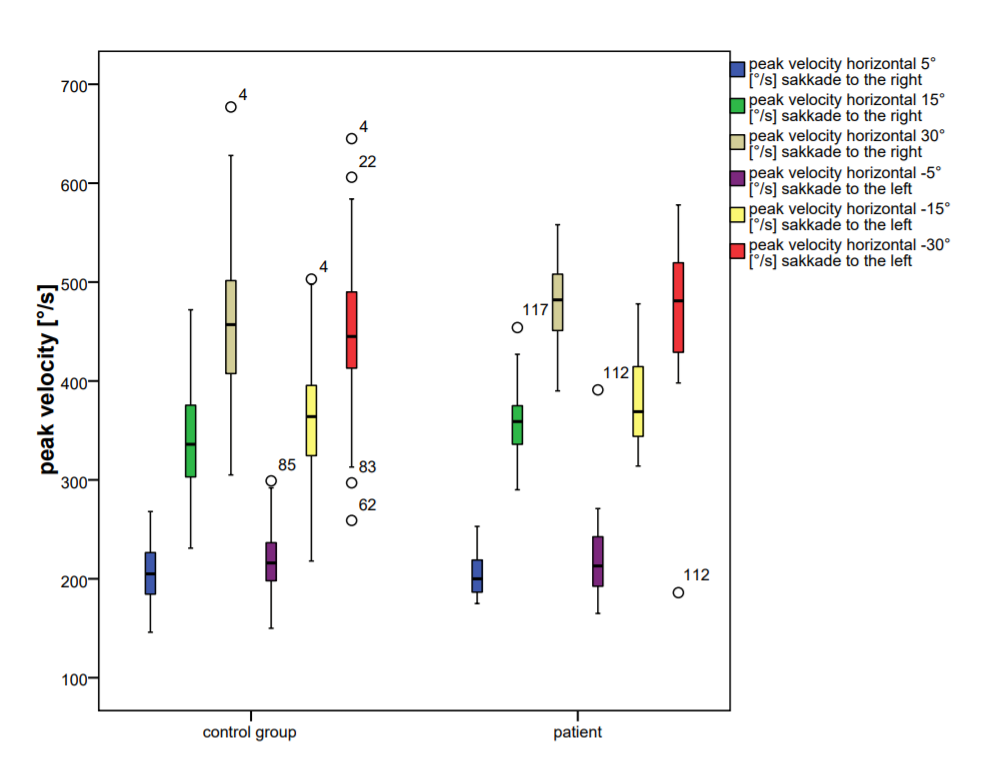
**

*Figure: Peak velocity (y-axis) is displayed for PKU patients vs. the control group for different target eccentricities. The whiskers extend to the minimum and maximum, when there are no outliers. Outliers are displayed as circles, meaning values defined by a distance of more than 1.5 times the interquartile distance from the box. The lower boundary of the box is the 25th percentile (25 % quartile), the line within the box indicates the 50th percentile (median) and the upper boundary represents the 75th percentile (75 % quartile). The peak velocity increases with increasing target eccentricity.*
