# Supplementary material for: Saccadic reaction time and ocular findings in phenylketonuria
Source: Orphanet J Rare Dis. 2020 May 25;15:124. doi: 10.1186/s13023-020-01407-7 (PMC7249436; doi:10.1186/s13023-020-01407-7)
Supplement: Supplementary file 5 — Additional file 5. Boxplots of gain (accuracy) in PKU patients and controls (Figure). [file 13023_2020_1407_MOESM5_ESM.docx]

**Additional file 5: Boxplots of gain (accuracy) in PKU patients and controls**


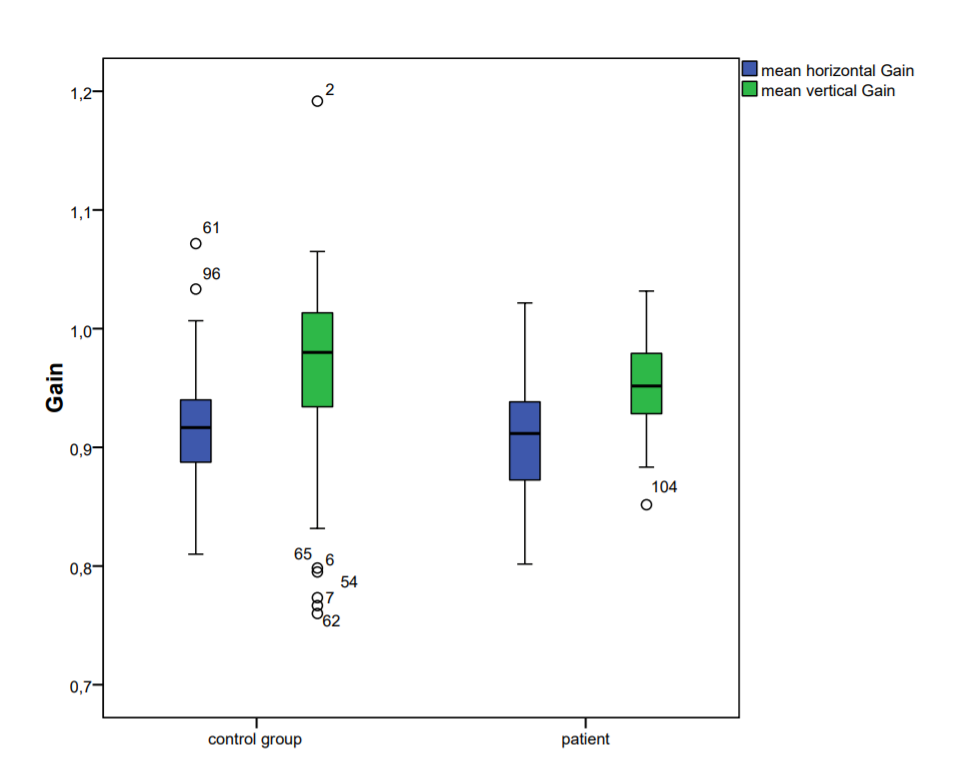


*Figure: Gain (y-axis) is displayed for PKU patients vs. the control group for horizontal (blue) and vertical (green) saccades. Most saccades displayed were isometric (i.e. between 0.9 and 1.1) or slightly hypometric (<0.9); the vertical saccades were more hypometric than the horizontal ones in both the PKU patients and the controls.*
